# Improvement of Lightweight Convolutional Neural Network Model Based on YOLO Algorithm and Its Research in Pavement Defect Detection

**DOI:** 10.3390/s22093537

**Published:** 2022-05-06

**Authors:** Fu-Jun Du, Shuang-Jian Jiao

**Affiliations:** Department of Civil Engineering, College of Engineering, Ocean University of China, Qingdao 266100, China; dufujun@stu.ouc.edu.cn

**Keywords:** pavement defects, deep learning, convolutional neural network, YOLOv5S, automated inspection, embedded equipment

## Abstract

To ensure the safe operation of highway traffic lines, given the imperfect feature extraction of existing road pit defect detection models and the practicability of detection equipment, this paper proposes a lightweight target detection algorithm with enhanced feature extraction based on the YOLO (You Only Look Once) algorithm. The BIFPN (Bidirectional Feature Pyramid Network) network structure is used for multi-scale feature fusion to enhance the feature extraction ability, and Varifocal Loss is used to optimize the sample imbalance problem, which improves the accuracy of road defect target detection. In the evaluation test of the model in the constructed PCD1 (Pavement Check Dataset) dataset, the mAP@.5 (mean Average Precision when IoU = 0.5) of the BV-YOLOv5S (BiFPN Varifocal Loss-YOLOv5S) model increased by 4.1%, 3%, and 0.9%, respectively, compared with the YOLOv3-tiny, YOLOv5S, and B-YOLOv5S (BiFPN-YOLOv5S; BV-YOLOv5S does not use the Improved Focal Loss function) models. Through the analysis and comparison of experimental results, it is proved that the proposed BV-YOLOv5S network model performs better and is more reliable in the detection of pavement defects and can meet the needs of road safety detection projects with high real-time and flexibility requirements.

## 1. Introduction

It is understood that the United States has the largest highway network in the world. As of 2019, the total mileage of the U.S. highway network reached 6,853,024 km, of which about 63% has been paved. By 2020, the total mileage of China’s highway network reached 5,198,000 km, of which 95% has been paved [1,2]. As more and more roads are paved, the maintenance of road pavements is a serious problem faced by the road maintenance departments of various countries. Pavement structural damage is the main cause of pavement defects. Once pavement cracks are formed, rainwater will accelerate the expansion of defects and create traps for moving vehicles, becoming one of the causes of car accidents. According to relevant data, from 2013 to 2016, a total of 11,386 people lost their lives due to road defects in India, which means that on average, about seven people die every day in India due to road surface defects [3]. In the United States, about half of the fatal car accidents that occur each year are caused by poor road maintenance. An 18-month study conducted by the Pacific Institute for Research and Evaluation examined information from the National Highway Traffic Safety Administration, because road defects, road icing, and other problems caused at least 42,000 Americans to lose their lives each year [4]. Pavement defects have introduced huge safety hazards to people’s travel. Therefore, to protect people’s lives and to provide for property safety, one of the important tasks of road safety and road maintenance is to discover and master road pavement defects in a timely manner.

In addition, highway engineering pavement defect detection technology is the most basic method of quality control in the construction stage of the life cycle of highway projects [5]. That technology is an important source of reference data for construction quality inspection, quality supervision, and quality control of highway engineering projects. It is also an important basis for engineering design parameters, construction quality control, construction completion acceptance, maintenance management, etc. 

Pavement is the first part of the highway structure to be contacted by an external force. It bears the pressure of the passing vehicle loads and is also affected by other indirect factors, such as temperature change, corrosion, and human damage [6]. The damage to highway structures is often first manifested at the surface, so the quality of surface structure directly affects the quality of the entire life cycle of highway engineering. As an important component quality control in highway engineering construction, road engineering pavement defect detection technology has great significance for scheduling control and cost control during the life cycle of highway engineering. The detection of pavement defects does not essentially reduce the costs of highway engineering; however, through testing, a scientific and correct assessment of a highway project can be carried out reasonably to enhance maintenance, reduce the rate of accidents that are due to poor quality, reduce the costs of design, and prepare for preventive management and control to extend the service life of highway projects [7]. Therefore, research on the detection technology of pavement defects in highway engineering is of great importance.

The automatic detection of pavement defects is of great significance in the quality assessment of asphalt pavements [8]. In the assessment specifications of asphalt pavement in many countries, the assessment of pavement quality is determined according to the road defect condition. For example, in China, according to the MQI (Maintenance Quality Indicator) index of asphalt roads, the technical condition of roads is divided into five grades [9]. Pavement condition assessment is time-consuming and laborious repetitive work for humans, but it is simple and easy-to-operate work for computers. Therefore, evaluating road conditions by using computer vision technology can save significant labor costs and improve evaluation speed, while avoiding human subjective factors, to generate different evaluation criteria.

At present, there are three main methods in the detection of road surface defects: manual inspection, automatic detection, and image processing technology. In developing countries, the inspection of highway pavements is usually completed by manual inspection [10]. Traditional manual inspection has resulted in poor safety, low efficiency, and high costs, and is susceptible to subjective factors, resulting in different standards of judgment. 

Defect detection of road pavement has been gradually replaced by automated equipment, such as inspection vehicles equipped with infrared or sensor equipment [11,12]. However, due to the complex characteristics of pavement defects and the road surface environment, automatic equipment brings certain difficulties to the detection of pavement defects, and presents difficulties in meeting the needs of actual engineering in terms of recognition accuracy and speed; in addition, the cost of automatic equipment is high and, accordingly, the cost of automatic detection is high. 

Image processing technology has the advantages of low cost and high efficiency, and precision is gradually improved with the development of technology. Therefore, many researchers use image processing technology to detect road surface damage [13,14,15,16,17]. Traditional image processing techniques usually use manually selected features, such as color [18], texture [19], and geometric characteristics [20], to segment pavement defects, and then perform a classification and matching of machine learning algorithms to achieve the detection of pavement defects. However, given the complexity of road environments, the traditional image processing method cannot meet the requirements of model generalization ability and robustness in practical engineering through artificially designed feature extraction. Compared with traditional image processing technology, image processing technology based on deep learning theory has higher precision faster speed, and strong embeddability [21,22] in the detection of pavement defects, etc. 

Computer vision technology based on deep learning has been widely used in the study of pavement defect detection. Hoang et al. [23] applied automatic detection technology to the investigation phase of asphalt pavement, using computer vision technology to detect repaired and unrepaired potholes, but its generalization ability was low. The technology needs further improvement if is to be applied to practical projects. Riid et al. [24] improved the detection performance of the model by manually selecting a deep convolutional neural network orthogonal frameset for training and digitizing, but the factors considered were too idealistic to be applied to actual highway engineering. Nguyen et al. [25] realized the automatic detection of cracks and potholes through the VGG16 network, and improved the robustness of the model through data enhancement processing technology, but the network structure of this model was large and it was difficult to meet the flexibility of embedded device applications. Maeda et al. [26] proposed the use of SSD Inception V2 and SSD MobileNet for road damage detection. The experimental results showed that the optimal model could achieve acceptable accuracy (77%) and recall (71%), outperforming conventional image processing techniques. Although the SSD network model can address inaccuracy in the detection of pavement defects, its speed is not as good as that of the YOLO series network in the detection of pavement defects. 

Ping et al. [27] conducted experiments on the performance of YOLO, SSD, HOG with SVM, and Faster R-CNN network models for pavement defect detection; they constructed a dataset of pavement defects and then used different models for comparison The experimental results showed that the YOLOv3 model of the YOLO network algorithm series had the best performance in the detection of road surface defects, with fast speed and reliable detection results. Du et al. [28] used the YOLOv3 algorithm to construct a pavement defect detection model to achieve automatic feature extraction, and the detection speed was increased, but the flexibility was still poor, and it was difficult to meet the flexibility required by embedded systems. Liu et al. [29] used the combination of 3D ground-penetrating radar and the YOLO algorithm to detect damage to pavement structures. This method explored the damage in the deep structure of pavement through radar, which can detect potential structural damage in advance, but the cost was high. Park et al. [30] used YOLOv4 to carry out defect detection research for pavement potholes, but their research only focused on the ground penetration of pavement pothole defects, without more challenging crack detection studies. Baek et al. [31] improved the detection speed of the model by processing images of road pits in grayscale and then inputting them into the YOLO detection mode, and achieved good performance. To a certain extent, the processed images reduced the amount of information, resulting in the reduction of the generalization ability and robustness of the model. Pena-Caballero et al. [32] proposed deploying the YOLOv3 algorithm to an embeddable device to detect pavement defects, but the experimental results showed that due to the large network structure of the YOLOv3 network model, the performance of real-time detection in embedded systems needs to be improved. Ahmed [33] used YOLOv5, YOLOR, and Faster R-CNN deep learning network models in detecting pavement defects. The resulting analysis showed that the YOLOv5 model is extremely flexible and suitable for real-time detection scenarios of embedded devices. However, in terms of accuracy (mAP@0.5: 58.9%), further improvement was needed.

The prospects for the application of target detection technology based on deep learning theory in highway pavement defect detection are very considerable, but there are still some difficulties, including the following: (1) The existing pavement defect dataset is insufficient, and the field collection of data is dangerous, low in inefficiency, and high in cost. (2) The road environment is easily affected by passing vehicles and roadside greening facilities, which cause uneven light intensity, bringing difficulties to the target detection model. (3) Some pavement defects are small in size, large in number, and easily missed, and the target detection model has low detection accuracy for pavement defects [33]. To solve these problems, in this paper we adopt a new strategy to build a PCD1 dataset on pavement defects. Inspired by the YOLOv5 [34] model, we propose the BV-YOLOv5S deep learning network model, which improves the YOLOv5 model’s performance. The feature extraction network adopts the BiFPN network structure [35] to strengthen the feature extraction network, which aims to mine deep-level information about pavement defects in an image, improve the feature extraction performance of the model, and reduce missed detection caused by lighting and size. Further, based on the BV-YOLOv5S deep learning network model, Varifocal Loss [36] will be used to replace Focal Loss, so that the attention of model training is biased toward high-quality samples, making full use of the effective information in the dataset, and further improving the detection accuracy of the model.

The rest of this paper is organized as follows: (1) In Section 1, we introduce the current status of pavement defects and existing pavement defect detection methods. (2) The improved method of lightweight convolutional neural network for pavement defects is introduced in detail in Section 2. (3) In Section 3, we present the details of the experiment. (4) In Section 4, we discuss a comparison of the experimental results. (5) Finally, in Section 5, we summarize our work.

## 2. Methods

### 2.1. Introduction to Algorithm and Network Structure

YOLOv5S is a target detection method based on deep learning theory. It mines the data features in samples through a deep network structure [37], making the trained model more suitable for detection of targets with complex features, such as road defects, which can be detected by transfer learning [38]. Transfer learning quickly completes the training of network models and deploys them to target detection tasks in different backgrounds. Target detection work is an important branch in the field of computer vision [39]. The target detection network based on deep learning theory is mainly divided into two categories, according to the generation of candidate frames: the single-stage target detection network, without candidate frame generation, and the two-stage object detection network for box proposal generation. Typical single-stage detection networks include the YOLO [40] series networks and the SSD [41] series networks. The main operating principle of the single-stage target detection network is dividing the image into small squares, each of which has a preset fixed anchor. Then, the objects in the picture are divided into different small squares for classification, and the target detection work of the image to be tested is completed. 

The two-stage target detection algorithm includes Fast RCNN [42], Faster RCNN [43], SPPNet [44], Mask R-CNN [45], and other network algorithms. The network principle is mainly that a large number of Windows are generated in the first stage. Windows uses a binary classification method to distinguish foreground and background. In the second stage, the region of interest (ROI) of target detection is used to deduct features from the feature map extracted by the convolutional neural network, and then the classification is performed again, which is different process compared to that of the first stage. The second stage of classification work is multi-target classification, which is used to distinguish the categories of different targets and to predict the position of an object by regression. 

The accuracy of the single-stage detection network is limited, due to the imbalance of the samples. However, with the introduction of Focal Loss, the accuracy of the single-stage network target detection network has been greatly improved [46], and the single-stage network has an absolute advantage in terms of speed. Therefore, the single-stage detection network has been applied on a larger scale in the field of real-time detection, and the lightweight model based on the YOLO network has shown great flexibility in the deployment tests of embedded devices [47].

**Figure 1:**
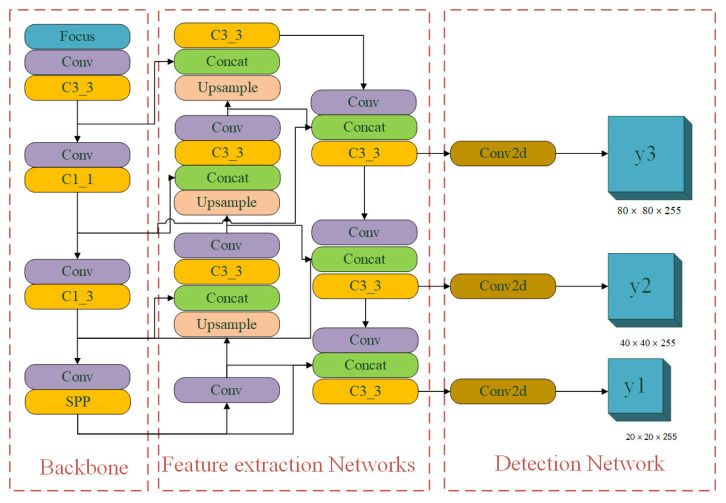
BV-YOLOv5S network structure. It is mainly composed of three parts: backbone networks, feature extraction networks, and detection networks.

With the large-scale application of GPU and the rapid development of computer vision technology, the target detection technology based on deep learning theory has an absolute advantage in performance compared with traditional image processing technology [48,49]. At present, highway pavement defect detection has become an important topic in the field of computer vision technology. Single-stage object detection networks can be effectively transferred to highway pavement defect detection through transfer learning. Compared with other object detection algorithms, YOLOv5S has the advantages of fast detection speed, high accuracy, and flexibility [24]. However, when it is applied to the detection of road pavement defects, there are still problems, such as difficulty in identification due to the small defect target, a high missed detection rate, and difficulty in mining the deep-level information of a defect image. To solve these difficulties, this paper proposes the BV-YOLOv5S network model, the network structure of which is shown in Figure 1, The network structure is mainly composed of backbone networks, feature extraction networks, and detection networks. To further improve accuracy, the BV-YOLOv5S deep learning network model uses Varifocal Loss to solve the sample imbalance problem in the single-stage target detection network model.

**Figure 2:**
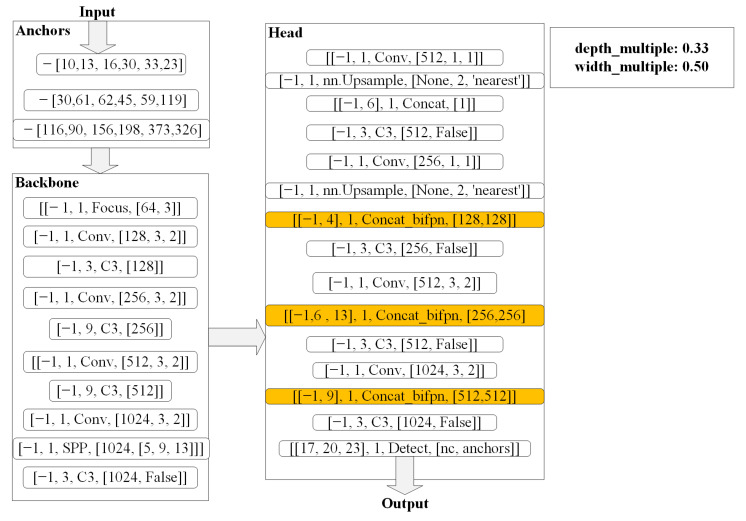
The detailed processing flow of the BV-YOLOv5S model to realize road defect detection. (Depth_multiple and width_multiple are the depth and width of the network, respectively. The anchor part is the size setting of the anchor. The content expression format of the backbone part [number, module, args] were from the input of the layer, where number is the number of the layer, module is the class name, and args is the initialization parameter of the class. The head part is the same as the backbone part in terms of content expression format).

To present the enhanced feature extraction network designed in this work in a more complete and detailed manner, we have drawn a detailed processing flow chart for the BV-YOLOv5S model to achieve road defect detection. Through the model’s processing flow chart, we express the main work of this paper in detail, comprehensively and intuitively, as shown in Figure 2. In Figure 2, we divide the implementation process of the pavement defect detection algorithm into three parts: anchor, backbone, and head. We mainly redesigned the network structure for the head layer, in which the color deepened in the BifPN network structure, and an enhanced feature extraction network as shown in Figure 2 is constructed. Regarding the detailed network structure of BiFPN, we will introduce it in detail in Section 2.2.

### 2.2. Feature Extraction Networks of the BV-YOLOv5S Model

**Figure 3:**
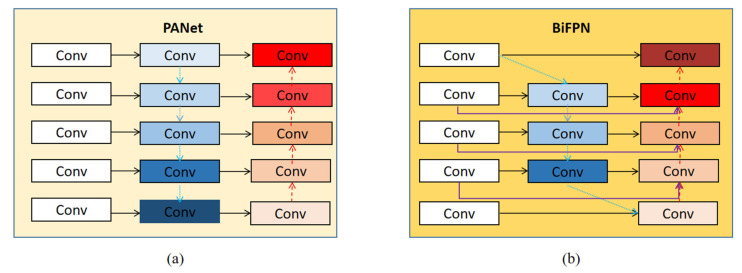
Comparison of BiFPN and PANet network structures [35,51]. (**a**) is the PANet network structure, and (**b**) is the BiFPN network structure, where conv is convolution node.

**Figure 4:**
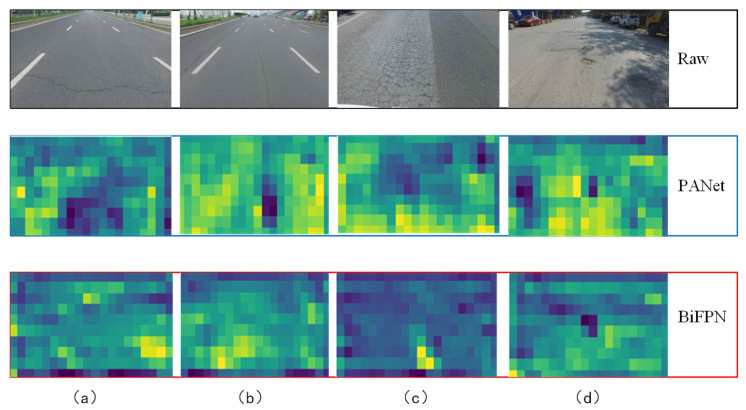
Feature extraction diagram of four types of pavement defects by BiFPN and PANet network structures. (**a**) lateral cracking, (**b**) longitudinal cracking, (**c**) alligator cracking, and (**d**) pothole.

In the deep learning network model, with the continuous deepening of the number of network layers, each layer of a network will cause the loss of information to a certain extent and lead to the loss of features. By fusing multi-scale feature fusion networks, the detection accuracy of the model can be improved [50]. The feature extraction network of the YOLOv5S model adopts the PANet network structure. Focusing on the specificity of pavement defects, especially the characteristics of small pavement cracks, the model excavates deeper information about pavement defects shown in the picture. We improved the original feature extraction network, PANet, into a BiFPN network structure. It aims to enhance the depth of information mining and to further improve the feature extraction capability of the model. The network structures of BiFPN and PANet are shown in Figure 3. As shown in Figure 3b, the BiFPN structure diagram, the blue arrow is the top-down path, which transmits the semantic information of high-level features, the red arrow is the bottom-up path, which transmits the location information of low-level features, and the purple part is an additional path when the input point and output point are located in the same layer, to fuse more features. Figure 4 is a visualization of the feature extraction of four types of road surfaces by PANet and BiFPN.

To enhance the transmission of image features of the deep learning model, we adopted the BiFPN network structure to strengthen the feature extraction ability of the deep learning network, compared with the original feature fusion network, PANet, as shown in Figure 3a, eliminating single-sided input nodes with less contribution to fusion. As shown in Figure 3b, the BiFPN network strengthens higher-level feature fusion in the processing path, processing each bidirectional path (top-down and bottom-up) as a feature network layer, and repeats this process multiple times in the same layer. Through the fusion of weighted features, the importance of different input features is learned, and differentiated fusion is carried out for different features. BiFPN uses fast normalized fusion to fuse weighted features, which are defined as in Equation (1). The learning weight, wi, uses the ReLU activation function, with a value of ε = 0.0001, to strengthen the stability of the value. To further improve the detection efficiency of the deep network learning model, BiFPN uses separable convolutional fusion features and adds batch normalization and activation after each convolution. We took layer 6, as shown in Figure 4b, as an example and described the definitions of two fusion features as shown in Equation (2).

(1) $$O=\sum_{i}\frac{w_{i}}{\varepsilon +\sum_{j}w_{j}}\;\cdot I_{i}$$

(2) $$P_{6}^{td}=Conv\;\left(\frac{w_{1}\cdot P_{6}^{in}+w_{2\;}\cdot Rwsize\;\left(P_{7}^{in}\right)}{w_{1}+w_{2}+\varepsilon }\right)\;P_{6}^{out}=Conv\;\left(\frac{w_{1}^{'}\cdot P_{6}^{in}+w_{2}^{'}\cdot P_{6}^{td}+w_{3}^{'}\;\cdot \;Resize\;\left(P_{5}^{out}\right)}{w_{1}^{'}+w_{2}^{'}+w_{3}^{'}+\varepsilon }\right)$$

where $P_{6}^{td}$ represents the middle feature of the sixth layer from top to bottom and $P_{6}^{out}$ is the output feature of the sixth layer from the bottom up. Through the interconnection and fusion between different layers, BiFPN’s bidirectional cross-scale connection and fast normalization fusion are finally realized.

### 2.3. Improved Focal Loss Function of BV-YOLOv5S Model

Existing detectors rank NMS detections by predicting an additional IoU score [52] or a center score [53] as an indicator incorporated into the prediction criteria, which can alleviate the error between a classification score and localization accuracy. However, the standard obtained by multiplying two imperfect scores may produce larger errors, and experiments have shown that this is not a perfect practice in the field of object detection [36]. In this paper, we determined an IoU-aware classification score as the target presence confidence by simultaneously predicting the perceptual classification score (IACS), representing the localization accuracy of a specific object class to be detected; we also determined the generated bounding box by incorporating additional predictions into the classification score, for a joint representation of the localization accuracy. The YOLOv5 deep learning network model uses Focal Loss to deal with the imbalance of positive and negative samples in the target detection of the YOLOv5 model. Its definition formula is shown in Equation (3), where α is the lost weight and pγ is the weight of different samples; the sample weight increases for samples that are difficult to classify, reducing the impact of easy-to-classify samples on the loss function. The model pays more attention to the training of samples that are difficult to classify, but creates positive and negative samples equally; thus, it does not put the focus of training on high-quality samples. Given the complex background of pavement defects, the effective features in the sample images are difficult to highlight. In the BV-YOLOv5S deep network model, we used Varifocal Loss to deal with the problem of sample imbalance and to increase the weight of positive sample losses with high IoU values. 

Focusing training on high-quality samples increases the robustness of the model. Its definition formula is as in Equation (4), where p is the predicted Iou-aware Cls_score (IACS) and q is the target IoU score. For the positive sample q in training, it is the IoU between the b box and the gt box, and for the negative sample q in the training, the value is 0.

(3) FLp, y=−α1−plγogp−1−αplγog1−p  if y=1otherwise  

(4) VFLp, q=−qqlogp+(1−qlog1−p)−αplγog1−p  q <0q=0  

Compared with other target detection objects, pavement defect images have the characteristics of complex backgrounds and diverse shapes; there is a high false detection rate during recognition, and the preparation of high-quality datasets raises problems of low efficiency and high cost. To solve these problems, we used Varifocal Loss instead of Focal Loss for calculation, predicting IACS and classification scores. At the same time, we focused on high-quality samples for training, and for efficiently we used the information in the dataset.

In this paper, our detailed improvement steps for the sample imbalance function, Focal Loss are as follows: first, according to the source code of Varifocal Loss [40], the applicability of the source code was converted to make the source code conform to the network structure of the YOLO algorithm; then, the original sample imbalance processing function was replaced to complete the construction of the BV-YOLOv5S road surface defect detection model.

## 3. Experiment

### 3.1. Experiment Environment and Evaluation Index

#### 3.1.1. Experiment Environment

The model proposed in this paper was tested under laboratory conditions and the results were analyzed quantitatively and qualitatively. The deep learning model development tool used was Anaconda3, and the deep network learning model development hardware and environment in the laboratory were CPU: Intel(R) Xeon(R) Gold 5218; GPU: GeForce RTX 2080 Ti/11 GB; system: ubuntu18.04, CUDA10.2 with model acceleration training function, cuDNN7; pytorch1.7.0 was used as the training framework of the model.

#### 3.1.2. Evaluation Metrics

To verify the performance of our proposed BV-YOLOv5S network model in pavement defect detection, a confusion matrix evaluation index was introduced to evaluate the model. The confusion matrix contains four types of definitions: TN (predict negative samples as negative samples), FN (predict positive samples as negative samples), TP (predict positive samples as positive samples), and FP (predict negative samples as positive samples).

(1) Precision, Recall, F1-score evaluation indicators

To comprehensively evaluate the performance of the model in the PCD1 dataset, we introduced quantitative analysis indicators: precision, recall, and F1-score as defined in Equations (5)–(7). Precision, recall, and F1-score have commonly used evaluation indicators for target detection algorithms, and they play an irreplaceable role in the evaluation of models.

(5) $$P=\frac{\mathrm{TP}}{\mathrm{TP}+\mathrm{FP}}$$

(6) $$R=\frac{\mathrm{TP}}{\mathrm{TP}+\mathrm{FN}}$$

(7) $$F1-\mathrm{score}=\frac{2\times P\times R}{P+R}$$

(2) Detection rate

Detection speed is a critical requirement for engineering practical applications, and we used frame rate (FPS) to show detection speed, which is an important metric for model evaluation. Generally speaking, if FPS is ≥30, it can basically meet the demand, and the video detection function of FPS ≥ 60 is smooth.

(3) PR curve, mAP@.5 evaluation indicators

The PR curve takes recall as the abscissa and precision as the ordinate to draw the curve. If the PR curve of one model could completely wrap up the PR curve of the other model, then the performance of the former model could be considered to be better. If it could not be judged directly, the comparison could be made according to the area under each model curve.

mAP@.5 means that under the condition of lOU = 0.5, the average value of precision is calculated for the four types of defects. The average value of precision is an important indicator in the model evaluation process. AP is calculated by precision and recall, and AP is defined as:

(8) $$AP=\frac{1}{11}\sum_{\gamma \in \left\{0,0.1,0.2\cdots ,1\right\}}\rho_{\mathrm{int}erp(r)}$$

When we calculated the AP of the four categories of defects under the condition of lOU = 0.5, we obtained the detected mAP@.5, which is defined as follows:

(9) $$mAP=\frac{\sum_{i=1}^{4}AP_{i}}{4}$$

### 3.2. Data Collection and Processing

**Figure 5:**
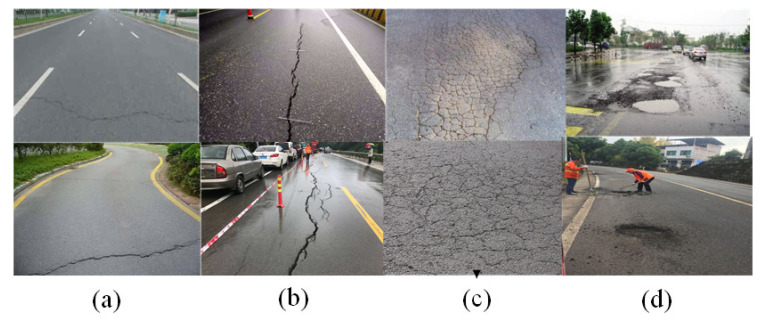
Partial image of four typical defect types in PCD1. (**a**) lateral cracking, (**b**) longitudinal cracking, (**c**) alligator cracking, and (**d**) pothole.

In this paper, we have constructed a pavement defect detection dataset—the PCD1 dataset. As far as we know, the current public datasets for defect recognition have different standards for such indicators as image shooting angle, light intensity, and clarity. The quality of such datasets is such that it is difficult to meet the requirements of use, so we decided to build our own defect dataset for road surfaces. Based on the existing public datasets, RDD2020 [54] and SDNET2018 [55], the quality of the PCD1 dataset was improved by using the Baidu Street View Map and web crawler technology, as well as field collection. The field acquisition device was the Huawei Mate30pro rear 40-megapixel camera. Some of the acquisition parameters were ISO = 50, F:1.6, S:1/1182 s, focal length: 27 mm, and shooting depression angle: 45°–60°. The PCD1 dataset was built based on diverse data, clear pictures, and a shooting angle of 45°–60°. To make full use of the dataset and to improve the generalization ability of the model, the images containing multiple types of defects were preferentially used. After careful selection, the collected images were standardized before model training, and the images were reduced to 640 × 640 size so that the YOLO model could exert the best training performance. After standardization, according to different types of pavement defects, labeling was used to manually label data. The file format of the labeling was txt, and a total of 5600 pieces of data were formed. Figure 5 shows four typical defect types in PCD1. According to the needs of the experiment, it was randomly divided into a training set, a validation set, and a test set, according to 6:2:2. The details of the defect distribution of the dataset are shown in Table 1.

### 3.3. Data Collection and Processing

We trained YOLOv3-Tiny, YOLOv5s, B-YOLOv5s, and BV-YOLOv5S, as proposed in this paper, in the same training set that was independent of the validation and test sets. The YOLOv3-Tiny and YOLOv5S algorithms are widely used in the field of defect detection, due to their strong flexibility, high accuracy, and fast speed, and they are advanced in the application of lightweight convolutional neural networks [56]. Many researchers in other professional fields have applied the BiFPN network structure to the YOLO algorithm series and achieved good target detection results [57,58,59]. For this reason, we established the B-YOLOv5S network and applied it to the detection of pavement defects. We further compared and evaluated the performance of the BV-YOLOv5S model proposed in this paper in pavement defect applications.

**Figure 6:**
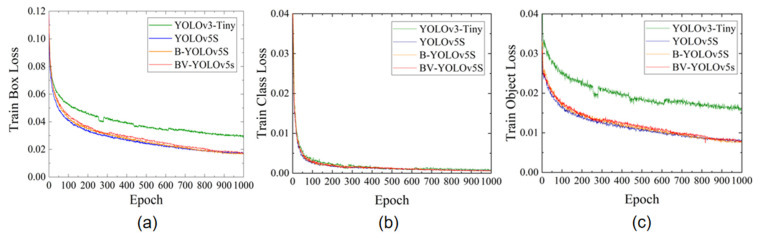
Comparison of training losses of four models. (**a**) train box loss. (**b**) train class loss. (**c**) train object loss.

We set the epoch to 1000 for the training of this study and used the cosine annealing method to adjust the learning rate. The initial learning rate was lr0 = 0.01, and the cyclic learning rate was lrf = 0.2, which was helpful for the model’s convergence loss. To apply the YOLO network structure, the size of all input images was 640 × 640, and the training process of the whole model took about 6 h. Loss represented the gap between the predicted value and the actual value. As the gap gradually decreased and converged, it meant that the model approached the upper limit of performance determined by the dataset. A comparison of the training loss function curves of the four models is shown in Figure 6.

As shown in Figure 6, the loss value fluctuates greatly at the beginning of training for each category, indicating that the initial hyperparameters we used were reasonable. After a certain number of iterations, the fluctuation of the loss curve gradually decreased, but as shown in Figure 6, we found that the convergence loss performance of YOLOv3-Tiny was poor. The loss function convergence performance of B-YOLOv5S and BV-YOLOv5S was similar, and better than the loss performance of YOLOv5S.

## 4. Results and Discussion

### 4.1. Evaluation Metrics

**Figure 7:**
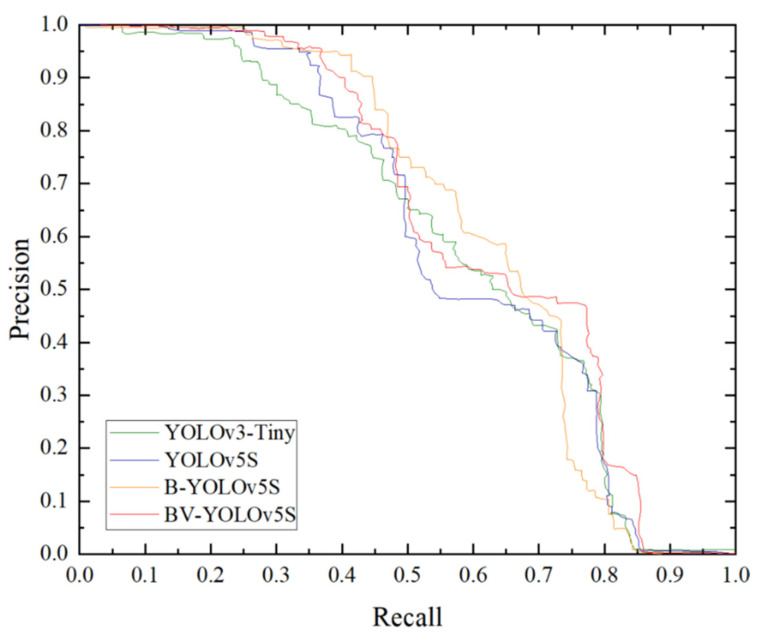
PR curves of four pavement defect detection models on the test set.

After the training of the four models, we used the test dataset independent of the training and validation sets to evaluate the models. During testing, we set IoU to 0.5 to divide the positive and negative samples, and we plotted the PR curves for the performance of different models. As shown in Figure 7, through the PR curve we could see that the performance of the improved YOLOv5S model was significantly better than that of YOLOv3-Tiny and YOLOv5S.

### 4.2. Discussion of Results

**Figure 8:**
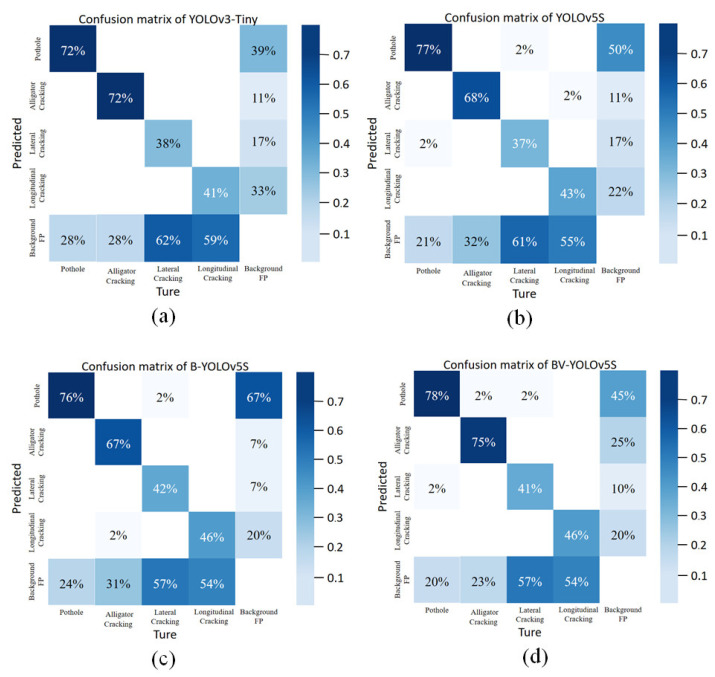
The confusion matrix of the comparison of the different defect detection algorithms. (**a**) YOLOv3-Tiny; (**b**) YOLOv5S; (**c**) B-YOLOv5S; (**d**) BV-YOLOv5S.

In Table 2, we summarize the performance of four deep learning network models in the PCD1 test dataset, and their confusion matrices are shown in Figure 8. From the confusion matrix, we found that BV-YOLOv5S was ahead or even far ahead of the other three models in the correct classification of pothole and alligator cracking, and was the same as B-YOLOv5 in the correct classification of longitudinal cracking and ahead of the other two models. In the correct classification of lateral cracking, although it was ahead of YOLOv3-Tiny and YOLOv5S, the correct rate was lower than that of B-YOLOv5S. Overall, the BV-YOLOv5S classification performance outperformed the other three models in the confusion matrix. However, for the defects of lateral cracking and longitudinal cracking, the classification effect of the four models was poor, resulting in a largely missed detection phenomenon. This phenomenon occurred because, for the deep network learning model, the small target of the crack, the variety of shape, and the uncertainty of the width caused certain difficulties in the feature information extraction of the target detection model, which was the difficulty in the field of small target detection. 

This phenomenon will be the focus of our future work. In the next step, we will enhance the accuracy of the model in crack detection by further improving the feature extraction ability of the object detection model network. 

Specifically, compared with the YOLOv3-Tiny model, the deep network learning model BV-YOLOv5S, the YOLOv5S model, and the B-YOLOv5S model mAP@.5 increased by 4.1%, 3%, and 0.9%, respectively. Precision increased by 12.7% and 0.5%, respectively, which was 1.2% lower than that of the B-YOLOv5S model; recall increased by 1.7%, 4.1%, and 2.9%, respectively. The F1-score increased by 5.5%, 3.1%, and 1.7%, respectively. As shown in Table 2, we found that the recall values of the four types of models were low, mainly due to the shallow network layers of the lightweight deep learning network model, The ability to extract features and learn was low, but this improved the running speed and flexibility of the model in meeting the needs of terminal deployment and practical engineering. We found that BV-YOLOv5S was significantly enhanced compared to YOLOv3-Tiny and YOLOv5S, in terms of speed, recall, and F1-score, which proved that our work is effective and can be used as a reference for other work in the defect detection field. According to the analysis of the experimental results of the detection speed, we found that the BiFPN network structure information processing speed was faster than that of the PANet network structure, which not only strengthened the feature extraction ability but also improved the detection speed of the model, and had better performance in real-time detection. However, the BV-YOLOv5S model adopted a more complex calculation method to deal with the problem of sample imbalance, which reduced the detection speed to a certain extent; nevertheless, it was sufficient for real-time detection requirements. In conclusion, our proposed BV-YOLOv5S deep network learning model comprehensively outperformed the YOLOv5S model in mAP@.5, precision, recall, F1-score, and its detection speed metrics went far beyond the current YOLOv3-Tiny network model, which is representative of lightweight target detection models.

Through comprehensive comparative analysis of mAP@.5, precision, recall, F1-score, and FPS evaluation indicators in the experimental results, we believe that the BV-YOLOv5S model is more robust in performance than the YOLOv3-Tiny, YOLOv5S, and B-YOLOv5S network models. The BV-YOLOv5S model proposed in this paper has higher accuracy and flexibility, and has stronger advantages for the deployment and practical application of embedded devices.

**Figure 9:**
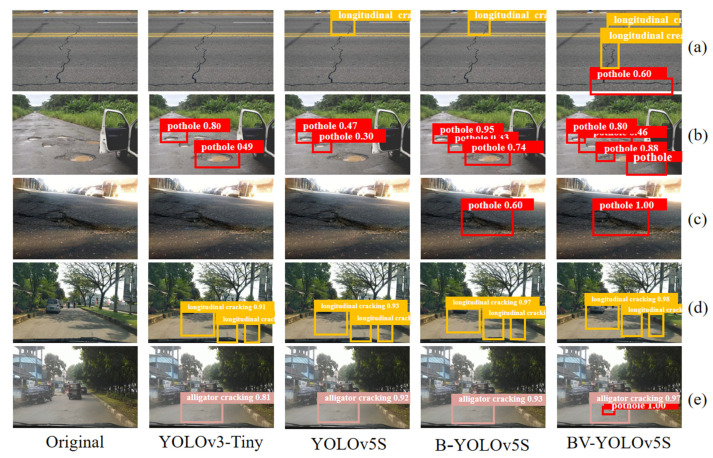
The test results of different network models in a complex environment. (**a**) The model detection results of four types of pavement defects in a rutting interference environment. (**b**) Detection results of four types of pavement defect models under the conditions of many and small targets. (**c**) The detection results of the four types of pavement defect models under environments of uneven illumination intensity. (**d**) The model detection results of four types of pavement defects under partial shadow occlusion conditions. (**e**) Alligator crack detection in the presence of small target potholes.

Due to the complex road surface environment, especially on rural roads, there are many defects in road surfaces. To comprehensively measure the performance of the BV-YOLOv5S model, we used small targets, multi-targets, and shadow-occluded targets to conduct visual experiments, as shown in Figure 9.

As shown in Figure 9a, we found that the detection performance of the YOLOv3-Tiny model is obviously disturbed by rutting and lane boundaries, and the anti-interference ability was poor. The detection performance of YOLOv5S and B-YOLOv5S was also interfered with by rutting and roadway boundaries to a certain extent, and BV-YOLOv5S had strong anti-interference ability in this environment. However, Figure 9a shows that the model was too sensitive to the detection of pavement potholes; it is necessary to further improve the generalization ability of the model in the future. 

As shown in Figure 9b, we tested the performance of the four defect models under the condition of multiple small targets, It can be seen from the figure that the YOLOv3-Tiny detection performance was average, and the small holes nearby were missed, which showed that the model was not sensitive to small targets, The performance of the YOLOv5S model was the worst; recent potholes were missed, and there were serious defects in performance. The B-YOLOv5S model had relatively good detection results. However, despite the missed detection of pits with inconspicuous defect characteristics in the vicinity and the missed detection of small targets at a distance, the BV-YOLOv5 model had the best performance in this detection. It could detect the pits in this range, showing an absolute advantage in performance. 

As shown in Figure 9c, the pavement defect detection performance of the four models was tested under the condition of uneven illumination intensity. It can be seen that the YOLOv3-Tiny and YOLOv5S networks were greatly interfered with by light intensity, resulting in obvious losses in detection performance. The B-YOLOv5S model could detect defects but had low confidence. The BV-YOLOv5S could accurately detect defects with a high degree of confidence. It was not interfered with by uneven light intensity and had a strong anti-interference ability with respect to the external environment. 

As shown in Figure 9d, the four models had similar detection capabilities under the condition of partial shadow occlusion, and a small part of the shadow did not have a great impact on the models. As shown in Figure 9e, we tested the model to detect alligator cracks under the interference of small target potholes. Through testing, we found that YOLOv3-Tiny had the lowest confidence, and the BV-YOLOv5S model had the highest confidence in alligator crack detection, and only this model could detect potholes for small objects. This shows that, to a certain extent, the handling of sample imbalance by Varifocal Loss enhances the model’s detection ability for small objects. It can be seen that by using BiFPN to enhance feature extraction, the detection accuracy of the model can be improved to a certain extent; in addition, by optimizing the sample imbalance processing method, the sensitivity of small target recognition can be improved, and the missed detection rate of targets with insignificant features can be reduced, with better performance in the detection of pavement defects. By further testing our improved model’s performance, we confirmed its performance advantages. The BV-YOLOv5S showed stronger practical advantages, compared with the YOLOv3-Tiny, YOLOv5S, and B-YOLOv5S models. In the quantitative evaluation results and the qualitative analysis, the BV-YOLOv5S model proposed in this paper showed strong anti-interference ability, high sensitivity to small targets, a low multi-target missed detection rate, and little influence of external environment interference, with good robustness and generalization.

In this experiment, we used asphalt pavement as the background of the defect detection model. Compared with cement pavement, the feature structure of asphalt pavement is more complex. Through the experiments in this dataset, we confirmed our results. At the same time, we could quickly carry out the training and deployment of the pavement defect detection model for cement pavement and other targets through transfer learning.

## 5. Conclusions

In this paper, an improved lightweight deep network learning model, BV-YOLOv5S, was proposed for the detection of asphalt road surface defects by embedded devices. First, we established a high-standard, high-quality PCD1 dataset employing public datasets, Baidu Street View maps, web crawler, and field photography. The data types of the datasets were enriched, and new data collection strategies contributed to the target detection datasets. Second, to realize the applicability of the model in embedded systems and to perform accurate detection under complex road conditions, we proposed a BV-YOLOv5S deep network learning model, based on the YOLOv5S deep network learning model. In the feature extraction network, the BiFPN network was used to replace the PANet network, with the aim of mining deeper information in the pavement defect images. The BV-YOLOv5S network model improved the Focal Loss and used Varifocal Loss as the loss function to deal with the imbalance problem between samples. More of the model’s attention was transferred to high-quality dataset samples, making full use of the effective information in the dataset. We trained and tested the proposed BV-YOLOv5S model and the YOLOv3-Tiny, YOLOv5S, and B-YOLOv5S network models under the same conditions. The experimental results showed that our proposed BV-YOLOv5S model in the mAP@.5 index improved by 4.1%, 3%, and 0.9%, respectively, compared with the deep network learning models YOLOv3-Tiny, YOLOv5S, and B-YOLOv5S. In addition, the recall and F1-score evaluation metrics were higher or even much higher than those of the B-YOLOv5S, YOLOv5S, and YOLOv3-Tiny models, O\outperforming YOLOv5S in detection speed and precision, and far exceeding YOLOv3-Tiny. The experimental results demonstrated that our proposed work is advanced.

Our proposed BV-YOLOv5S deep network learning model is most suitable for defect detection under actual road conditions and has good practicability and advancement. Compared with other object detection models, our model can be more flexibly deployed in mobile embedded devices, and compared with other lightweight object detection models, it has shown good performance in the tests. In subsequent work, the BV-YOLOv5S proposed in this paper will be applied to embedded devices in ordinary cars to further improve and realize the automatic detection of road surface defects.

## Figures and Tables

**Table 1:** Details of defect distribution in the PCD1 dataset.

Type of Dataset	Lateral Cracking	Longitudinal Cracking	Alligator Cracking	Pothole
Number	1350	1050	1400	1800

**Table 2:** Test results of different defect detection models in the pavement defect dataset. (Through independent test set testing, the precision, recall, and F1-score values of each category were provided and averaged).

Model	mAP@.5	Precision	Recall	F1-Score	FPS
YOLOv3-Tiny	0.594	0.737	0.573	0.646	167
YOLOv5S	0.605	0.859	0.549	0.670	238
B-YOLOv5S	0.626	0.876	0.561	0.684	278
BV-YOLOv5S	0.635	0.864	0.590	0.701	263

## Data Availability

The data presented in this study are available on request from the corresponding author. The data are not publicly available, as they involve the subsequent applications for patents, software copyright, and the publication of project deliverables.
